# Prenatal diagnosis to identify compound heterozygous variants in *PKDCC* that causes rhizomelic limb shortening with dysmorphic features in a fetus from China

**DOI:** 10.1186/s12920-023-01631-7

**Published:** 2023-08-17

**Authors:** Lulu Yan, Juan Cao, Yuxin Zhang, Yingwen Liu, Jinghui Zou, Biying Lou, Danyan Zhuang, Haibo Li

**Affiliations:** 1 The Central Laboratory of Birth Defects Prevention and Control, Ningbo Women and Children’s Hospital, Ningbo, Zhejiang 315000 China; 2 Department of Obstetrics, Ningbo Women and Children’s Hospital, Ningbo, Zhejiang 315000 China; 3 Department of Obstetrics, Ningbo Medical Center Lihuili Hospital, Ningbo, Zhejiang 315000 China; 4 Ningbo University School of Medicine, Ningbo, Zhejiang 315000 China

**Keywords:** RLSDF, *PKDCC* gene, Skeletal dysplasia, Whole exome sequencing

## Abstract

**Background:**

Rhizomelic limb shortening with dysmorphic features (RLSDF) has already been a disorder of the rare autosomal recessive skeletal dysplasia, just having a few reported cases. RLSDF is caused by protein kinase domain containing, cytoplasmic(*PKDCC*)gene variants. In this study, we describe the clinical features and potential RLSDF molecular etiology in a fetus from China.

**Methods:**

Genomic DNA (gDNA) extracted from the fetal muscle tissue and parents’ peripheral blood was subjected to chromosomal microarray analysis (CMA) and trio-based whole exome sequencing (Trio-WES). The candidate pathogenic variants were verified by using Sanger sequencing.

**Results:**

Trio-WES identified two compound heterozygous variants in *PKDCC*, c.346delC (p.Pro117Argfs*113) and c.994G > T (p.Glu332Ter), inherited from the father and mother, respectively. Both variants are classified as pathogenic according to American College of Medical Genetics and Genomics guidelines.

**Conclusions:**

We reported the first prenatal case of RLSDF caused by *PKDCC* in the Chinese population. Our findings extended the variation spectrum of *PKDCC* and emphasized the necessity of WES for the early diagnosis of skeletal dysplasia and other ultrasound structural abnormalities in fetuses.

## Background

Rhizomelic limb shortening with dysmorphic features (RLSDF, OMIM#618,821) is a rare autosomal recessive disorder characterized of rhizomelic shortening of the lower and upper limbs and changeable dysmorphic features, containing short neck, prominent forehead, macrocephaly, broad or depressed nasal bridge, micrognathia, along with long philtrum. Other features included obesity, mild plagiocephaly, laryngomalacia, short thumbs, mild bilateral conductive hearing loss, acanthosis nigricans, central hypotonia and mildly delayed myelination [1, 2]. This disease results from biallelic variants in the protein kinase domain containing, cytoplasmic (*PKDCC*) gene, which is located in chromosome 2p21 and contains seven exons. It encodes an integrated component of Hedgehog signaling demanded for normal chondrogenesis and bone development [2]. Studies on mice suggest that Hedgehog signaling can be regulated by *PKDCC* (also known as Vertebrate lonesome kinase, VLK) [3]. The Hedgehog signalling regulation is essential in the course of the bone development and repair [4]. *PKDCC* null mice show shorted long bones due to delayed endochondral ossification, as well as craniofacial abnormalities, including shortened and small nasal capsule and maxilla [5, 6].

To date, only nine families with molecularly confirmed variants of the *PKDCC* gene have been reported [1, 2]. The genotype-phenotype correlation of RLSDF is still unclear [2]. There is no specific treatment for RLSDF. The life expectancy of individuals with RLSDF is usually unaffected. In this study, we reported a female fetus with skeletal dysplasia through RLSDF, wherein we identified compound heterozygous variants affecting the *PKDCC* gene. To the best of our knowledge, this is the earliest identification of RLSDF by morphology ultrasound and the first report of *PKDCC* variants in a prenatal case of RLSDF.

## Materials and methods

### Clinical report

**Fig. 1:**
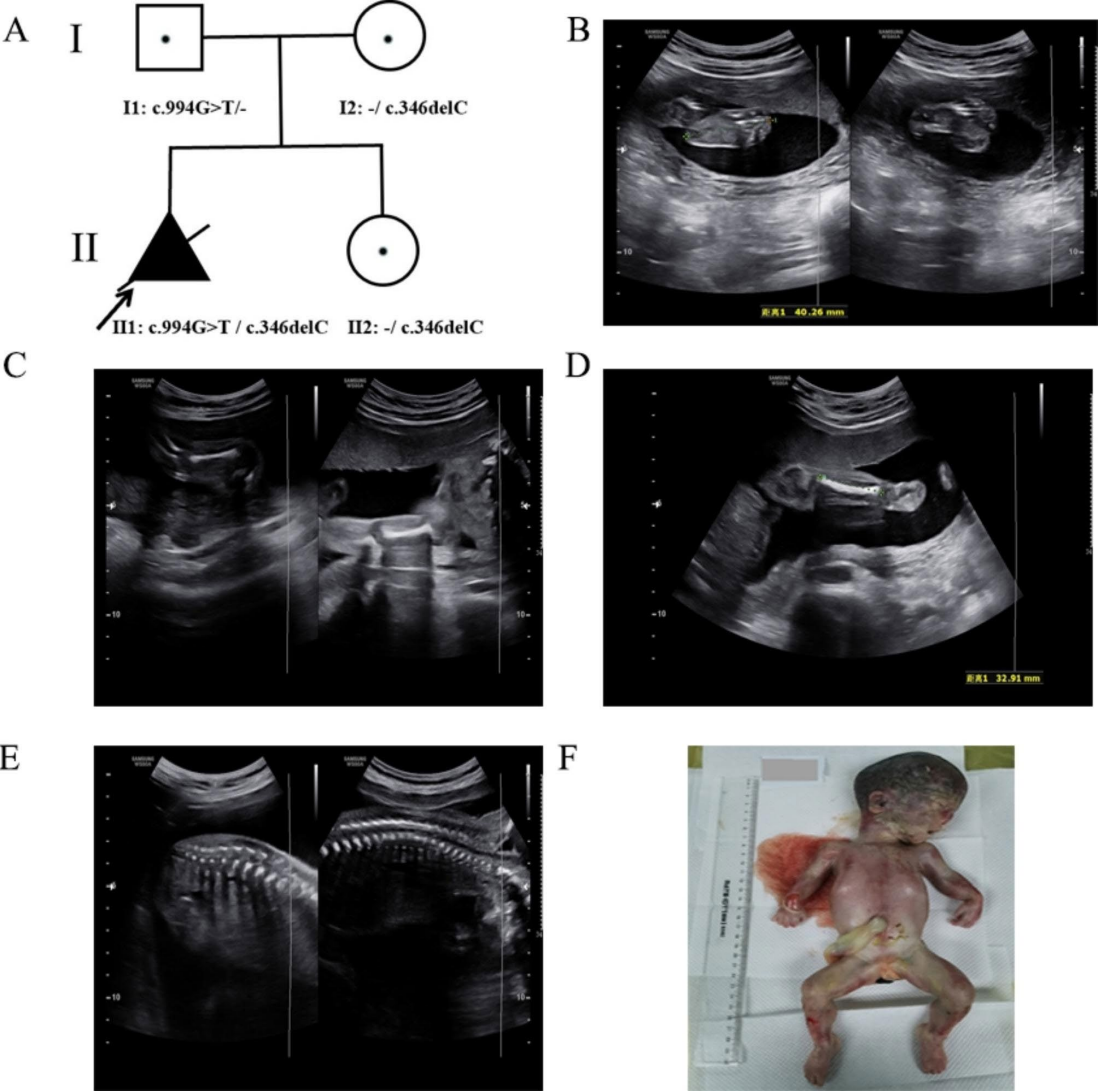
** A** Pedigree of the family with RLSDF. **B-E** Morphology ultrasound at a gestation of 22^+ 1^ weeks showing short humeri and ulna; the echoes of the spine and other bones were slightly weakened. **F** Appearance of the fetus after termination of pregnancy at a gestation period of 25^+ 3^ weeks, showing proband with rhizomelia of the upper limbs, flat face, prominent forehead, and nasal planus

The parents of the female fetus were nonconsanguineous and healthy. This was their first pregnancy (Fig. 1A). The pregnant woman conceived and was not exposed to poison or radioactive substances. The 22^+ 1^-week morphology ultrasound demonstrated rhizomelia of the upper limbs; the echoes of the spine and other bones slightly weakened (Fig. 1B-E). The length of the humerus and ulna was 27 mm (Table 1). An assumed diagnosis of skeletal dysplasia was made. Nevertheless, an accurate diagnosis was challenging to fulfill based upon the limited ultrasound discoveries. No other abnormalities were observed. The pregnant woman and her husband both showed no family history of skeletal dysplasia disorders or congenital malformations. Based on the ultrasound findings, the pregnancy was terminated 3 weeks later. The female fetus weighed 750 g with a height of 23 cm. Dysmorphic features were apparent with rhizomelia of the upper limbs, prominent forehead, and nasal planus (Fig. 1F). As for the exterior examination, its remainder was normal. Chromosomal microarray analysis (CMA) and trio-based whole exome sequencing (WES) analysis were conducted using the muscular tissue of the fetus and parents’ blood.

All participators offered the written informed consent. The protocols of this study were approved by the ethics committee of the Ningbo Women and Children’s Hospital (Zhejiang, China).

### Sample collection

Genomic DNA was extracted from the muscular tissue of the fetus and peripheral blood originating in parents using the QIAamp DNA Blood Mini Kit (QIAGEN, Germany) in light of the producer’s instructions (www.qiagen. com).

### Chromosomal microarray analysis

We conducted CMA using CytoScan 750 K array (Affymetrix, Santa Clara, CA, USA) according to the manufacturer’s protocol. Data was analyzed by the Chromosome Analysis Suite 4.0 (Afymetrix, Santa Clara, CA, USA). Copy number variants (CNVs) were further assessed according to the Online Mendelian Inheritance in Man (OMIM), ClinVar, Database of Genomic Variants, Database of Chromosomal Imbalance and Phenotype in Humans Using Ensembl Resources (DECIPHER), PubMed, and other databases. The pathogenicity interpretation of CNVs was conducted according to the standards and guidelines of the American College of Medical Genetics (ACMG) and the Clinical Genome Resource (ClinGen) [7].

### Whole exome sequencing (WES)

The WES of the genomic DNA of the proband and the parents was conducted with SureSelect Human All Exon V6 kits (Agilent) as well as a NovaSeq 6000 sequencer (Illumina, San Diego, CA). All indels and single nucleotide variants (SNVs) were filtrated and estimated through multiple databases, including the 1000 Genomes Project dataset, HapMap, Single-Nucleotide Polymorphism Database (dbSNP). Exome Variant Server databases were employed for the purpose of determining the pathogenicity and harmfulness of identified variations. All variants were subjected to biological effect analysis, that included the use of programs such as SIFT, PROVEAN, PolyPhen-2, Mutation Taster, deleterious annotation of genetic variants using neural networks (DANN), and Human Splicing Finder to forecast if an amino acid indel or substitution exerts a pivotal biological effect. Pathogenic variants are categorized in light of the ACMG and the Association for Molecular Pathology (AMP) [8].

**Sanger sequencing verification of the*PKDCC*gene**.

Sanger sequencing was conducted to confirm the potential causative variants from this family. Primers were devised by means of Primer 5.0. The primers are as follows: *PKDCC*-c.346delC, forward, 5′-TCCTCAACGTGCTCTTCGCTC-3′, reverse, 5′-GGGTTCTCTTCCAGCCAGGT-3′ and *PKDCC*-c.G994T, forward, 5′-AGAAGAGAAGTGCCAACCCC-3′, reverse, and 5′-TTCGATGGAGTTCCCGAGTC-3′. The sequence data was analyzed by means of Sequencing Analysis Software 6 (Applied Biosystems, Foster City, CA, USA).

## Results

**Fig. 2:**
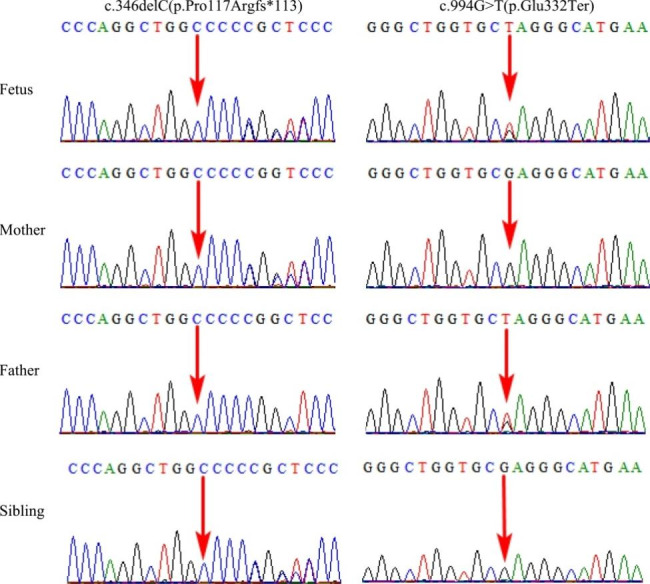
Sanger sequencing validation of the *PKDCC* variants identified by using WES. Variant c.346delC(p.Pro117Argfs*113) in the *PKDCC* gene in the heterozygous state (father and sibling). Variant c.994G > T(p.Glu332Ter) in the *PKDCC* gene in the heterozygous state (mother)

No abnormality was observed in the pathogenic CNVs in the fetus by CMA. WES revealed novel compound heterozygous variants in the *PKDCC* (NM_138370.3) gene c.346delC(p.Pro117Argfs*113) /c.994G > T(p.Glu332Ter). Neither variant was reported previously. The variant c.346delC(p.Pro117Argfs*113) of the fetus was a frameshift variant in exon 1 of the *PKDCC* gene, which could cause nonsense-mediated mRNA decay (NMD) in silico. We verified that this variant was inherited from her mother by Sanger sequencing. The other variant c.994G > T(p.Glu332Ter) of the *PKDCC* gene in the fetus was a nonsense variant and Sanger sequencing indicated that this variant was inherited from her father (Fig. 2). According to the classification guidelines for sequence variants from ACGM/AMP, the variants c.346delC (p.Pro117Argfs*113) and c.994G > T (p.Glu332Ter) in the *PKDCC* gene were classified to be pathogenic (PVS1, PM2_Supporting, PM3, PP4).

### Prenatal diagnosis

Following the identification of the *PKDCC* variants, the parents selected for prenatal diagnostic testing through amniotic fluid sampling at a gestation period of 19^+ 2^ weeks in their second pregnancy. That fetus was found to only carry the maternal variant (Fig. 2), consistent with the normal ultrasound scan at 19-weeks. At birth, there was no evidence of RLSDF and after 1 year, the girl child shows normal development.

## Discussion

Fetal skeletal dysplasia(SD) is one category of rare heterogeneous genetic disorder with 2.3 to 4.5 per 10,000 births [9]. In the 2019 revision of the Nosology and Classification of Genetic Skeletal Disorders, 461 types of hereditary skeleton diseases were identified, and are divided into 42 classes, containing 437 genes [10]. Most types of SD are the result of genetic variation, with a minority being associated with chromosomal anomalies, multisystem syndromes, or teratogenic environmental exposure [11]. However, the pathogenesis of more than half the SD remains unclear. Additionally, the clinical presentations of fetal SD are ambiguous, which results in a large challenge to diagnose accurately. Moreover, at present, an increasing number of molecular genetic approaches have already been adopted to prenatal diagnosis, and many diseases can be diagnosed by trio-WES based on next-generation sequencing technologies [12–15]. Prenatal genetic evaluation can provide parents more prospective information about the diagnosis, prognosis, and recurrence hazard for parents with the aim of making informed decisions and improve perinatal management [16].

In this study, we found that a female fetus with SD carried compound heterozygous *PKDCC* variants. We conducted WES on the fetus and her parents. We found that the compound heterozygous variants c.346delC(p.Pro117Argfs*113) and c.994G > T(p.Glu332Ter) of the *PKDCC* gene were in the sample of the fetus and they were inherited from her parents by Sanger sequencing, separately. This was the first reported case of RLSDF from China. Since the discovery of RLSDF at the year of 2018, 11 cases in total containing our patient have been reported [1, 2]. Most reported cases were linked to rhizomelia of the upper limb (100%), short stature (80%), hypertelorism (66.67%), flat face (66.67%), micrognathia (55.56%), short fifth digit (50%), hearing loss (50%), high and broad forehead (50%), macrocephaly (42.86%), sloping shoulders (33.33%), patellofemoral dislocation (33.33%), and prominent eyes (30%), rhizomelia of the upper limb was the most commonly reported (Table 2). Hypoplastic pituitary gland, growth hormone deficiency, and pectus excavatum were also observed in a small number of cases. In this study, the fetus showed dysmorphic characteristics and shortened long bones as the most evident morphological abnormalities. Therefore, the clinical phenotype of this fetus was basically consistent with the RLSDF. *PKDCC*-related SD is an extremely rare disorder. The leading skeletal characteristics of reported patients are similar, and variability was observed in the other detected phenotypes. Therefore, the prenatal evaluation of skeletal hypoplasia through ultrasound examination should lead to the consideration of genetic testing in the differential diagnosis.

**Table 1:** Bone length of the fetus at different gestational ages

Gestational age	Humerus	Ulna	Femur	Tibia
22^+ 1^ weeks	27 mm	27 mm	35 mm	33 mm
23^+ 1^ weeks	28 mm	27 mm	36 mm	33 mm

**Table 2:** Summary of all reported *PKDCC* variants leading to RLSDF

No.	Variant	Protein	Exon/Intron	Type	Zygosity	Skeletal findings	Craniofacial features	Other findings
Family 1 [1]	c.639 + 1G > T	UK	Intr1	Splice site	homozygous	Rhizomelic shortening and milder mesomelic shortening of the upper and lower extremities, Short fifth digit, bilateral middle finger clinodactyly, limited range of motion of shoulder joints, chronic joint pain, juvenile idiopathic arthritis and bilateral patellofemoral joint dislocation	Prominent forehead, downslanting palpebral fissures, broad nasal bridge, long philtrum	Obesity, left-sided headaches, acanthosis nigricans, chronic stage 1, kidney disease, a café au lait macule on upper right arm, depressed mood
Family 2 [1]	c.651 C > A	p.Tyr217Ter	Ex2	Nonsense	homozygous	Rhizomelia of upper limbs	Macrocephaly, short neck, micrognathia, mild proptosis, depressed nasal bridge, and long smooth philtrum	Short stature, diffculty gaining weight, central hypotonia, bilateral mild conductive hearing loss, laryngomalacia, poplyploid anal mass which had decreased in size, brisk deep tendonreflexes
Family 3 [2]	c.939dupA	p.Leu314Thrfs*29	Ex3	Frameshift	homozygous	Rhizomelic shortening of the upper limbs, mild coxa valga	Facial dysmorphism	-
Family 4 [2]	c.228dupG	p.Pro77Alafs*95	Ex1	Frameshift	homozygous	Rhizomelia of upper limbs	-	Mild neutropenia, hypoplastic pituitary gland on imaging, growth hormone deficiency and low TSH. mild hypertelorism and a slight metopic ridge
Family 5 [2]	c.606dupG	p.Leu203Alafs*96	Ex1	Frameshift	homozygous	Rhizomelia of upper limbs, bilateral clinodactyly	Thick, arched, eyebrows, nevus flammeus, thin upper lip	Widely spaced nipples, short stature, a long philtrum, hearing loss
Family 6 [2]	c.290_320del31/c.492delG	p.Leu97Profs*123/p.Leu165Serfs*65	Ex1/Ex1	Frameshift/ frameshift	compound heterozygous	Rhizomelia of the upper limbs	Midface hypoplasia, a high forehead, hypertelorism, a thin upper lip and a long philtrum	-
Family 7 [2]	c.785T > G	p.Leu262Arg	Ex3	Missense	homozygous	Rhizomelia, genuvarum, patellofemoral dislocation and short thumbs	Macrocephaly, high and broad forehead, flat face	Short stature, hearing impairment, hoarse voice
Family 8 [2]	c.639 + 1G > T/c.785T > G	UK/p.Leu262Arg	Intr1/Ex3	Splice site/missense	compound heterozygous	Rhizomelia and brachydactyly, sloping shoulders, pectus excavatum	Macrocephaly, prominent forehead, flat face, low set ears	Short stature
Family 9 [2]	c.380dup	p.Arg129Alafs*43	Ex1	Frameshift	homozygous	Rhizomelia	Prominent eyes/forehead, micrognathia, hypertelorism, cupped ears with rotated, fleshy ear lobes, an elfin-like face	Atrial septal defect, inferior vermis hypoplasia
In this study	c.346delC/c.994G > T	p.Pro117Rfs*113/p.Glu332Ter	Ex1/Ex3	Frameshift/nonsense	compound heterozygous	Rhizomelic shortening of the upper limbs	Flat face, prominent forehead, nasal planus	-

UK: unknown

**Fig. 3:**
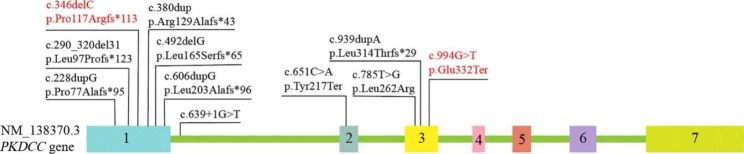
Summary of all variants in the *PKDCC* gene (NM_138370.3) associated with RLSDF. The reported variants are indicated in red (https://www.ncbi.nlm.nih.gov/clinvar/, the last access time was April 10, 2023)

The *PKDCC* gene was first reported by Imuta in 2009 [5]. Human *PKDCC* gene is 10.5 kb in size and encodes a secreted tyrosine-protein kinase with 493 amino acid residues [2]. The *PKDCC* is initially conveyed inside E-cadherin-positive cells however, is later limited inside E-cadherin-negative cells [6]. Currently, 11 variations in the *PKDCC* gene were reported to cause RLSDF in homozygous or compound heterozygous states, and the types of these variations varied, including frameshift (7/11), nonsense (2/11), missense (1/11), and splice (1/11) variants [1, 2]. This study investigated a fetus with compound heterozygous variants of *PKDCC*, namely, c.346delC(p.Pro117Argfs*113) and c.994G > T(p.Glu332Ter), that represent frameshift mutation and nonsense, respectively. This is consistent with the recessive inheritance pattern in this family and leads to the typical skeletal phenotype. Additionally, six of the *PKDCC* variants were located in exon 1, one of them in exon 2, three of them in exon 3, and one of them in intron 1 (shown in Fig. 3). We speculated that exon 1 may be a hotspot region and an important domain of the *PKDCC* gene. However, there is no clear correlation between genotype-phenotype of RLSDF.

*PKDCC* is localized in the Golgi complex and has been associated with the longitudinal skeleton development through the regulation of chondrocyte formation [6]. During the embryonic development of mice, *PKDCC* is mainly expressed in condensing mesenchymal cells, with high expression in limb buds and branchial arches [5, 6, 17]. In mouse embryos, homozygous *PKDCC* knockout mice show deficient long bone elongation, cranial abnormalities due to the delayed formation in the shortened intestine, sternal dysgraphia, flat proliferative chondrocyte, lung hypoplasia, and cleft palate. Additionally, the newborn knockout mice died from abnormal respiration a few hours after birth [5, 6]. The Glioma-associated oncogene homolog (GLI) family zinc finger 3 (*GLI3*) and the Indian hedgehog (*IHH*) genes are members of the hedgehog pathway in bone growth. Mice lacking these genes show skeletal abnormalities, including severe dwarfism of limbs, a short body length, and craniofacial abnormalities [18–21]. Notably, *PKDCC* and *GLI3* double knockout mice have more severe skeletal abnormalities than single knockout mice, indicating that *PKDCC* genetically interacts with *GLI3* and also affects bone growth development through the hedgehog pathway [22]. To date, the exact pathophysiological mechanism underlying RLSDF is still not understood.

One limitation of this study is the other characteristics (except for SD and facial dysmorphism) of the fetus could not be evaluated. Although the compound heterozygous variants may impact the protein stability, this should be confirmed by functional studies. Our results emphasize the need for prenatal diagnosis. WES is useful to recognize any associated genetic disorders and provide personalized care for patients with SD and other ultrasonic structure abnormalities.

In summary, our observation of this early phenotype should help in further elucidating the function of *PKDCC* and its role in skeletal development. Our results have significant reference value for the molecular diagnosis of RLSDF in future studies. Our study helped expend the *PKDCC* pathogenic variant spectrum of RLSDF. Further functional validation is necessary to elucidate the pathogenic mechanism of the *PKDCC* gene in RLSDF.

## Data Availability

The detected variants have been submitted to the LOVD, direct link: https://databases.lovd.nl/shared/individuals/00435461.

## Abbreviations

RLSDF: Rhizomelic limb shortening with dysmorphic features
CMA: Chromosomal microarray analysis
WES: Whole exome sequencing
CNVs: Copy number variants
SNVs: Single-nucleotide polymorphism database
SNP: Single-Nucleotide Polymorphism Database
ACMG: American College of Medical Genetics and Genomics
AMP: Association for Molecular Pathology
NMD: Nonsense-mediated mRNA decay
SD: Skeletal dysplasia
IHH: Indian hedgehog
GLI3: GLI family zinc finger 3
